# Supplementary material for: Chromothripsis during telomere crisis is independent of NHEJ, and consistent with a replicative origin
Source: Genome Res. 2019 May;29(5):737–49. doi: 10.1101/gr.240705.118 (PMC6499312; doi:10.1101/gr.240705.118)
Supplement: Supplemental Material [file supp_gr.240705.118_Supplemental_file_1.zip › contigs/annotated_contigs/DB91/contig.2.DB91_length_388_mean_cov_4.54381443299.docx]

**DB91_length_388_mean_cov_4.54381443299**

```
CATCTCCCCCCCTCCCCCCCCTCGTCCCCCCCCCCACCCC|CTGCCCCCCCCCCCCCCCCACGCCCCCCCCCCCCCCCCC|CTTCGCCC
                                         >chr14:101364088-101364127 - E=6e-03 p=0e+00
CTCCCCCCCTGCCGCC|ACCCCTCTGCCCCCGGTTCCCATCCCCCTTGGGCTGCTTGGTCCACACAGCAGTGTGGGGGCCAGTAGGCCC
                 >chr8:144580597-144580882 - E=3e-160
AAGGTGGTGAGTGGGCAGAACACGGGGTGACCCCCTTCAGCCGGGGAGGACAGGGCTGCATGGGGCAGGAGGCTGGTTGGGAGCACTGC

TGCCTACTTGTATGTCCTAGCACAGCTCTGGGGGCATCCTGGAGCCACCACAGCCTCCACGGCCCTTCCTCGCCTCCGTGGCAGGACAG

CAGGCTGGCCAGGCAAGATGACCGGTGAGCGGGCC
```
